# Synthesis and Characterization of Poly(Vinyl Alcohol)-Chitosan-Hydroxyapatite Scaffolds: A Promising Alternative for Bone Tissue Regeneration

**DOI:** 10.3390/molecules23102414

**Published:** 2018-09-20

**Authors:** Sergio Pineda-Castillo, Andrés Bernal-Ballén, Cristian Bernal-López, Hugo Segura-Puello, Diana Nieto-Mosquera, Andrea Villamil-Ballesteros, Diana Muñoz-Forero, Lukas Munster

**Affiliations:** 1 Grupo de Investigación en Ingeniería Biomédica, Vicerrectoría de Investigaciones, Universidad Manuela Beltrán, Avenida Circunvalar No. 60-00, Bogotá 110231, Colombia; sergiopinedac@outlook.com (S.P.-C.); bernalip10@hotmail.com (C.B.-L.); 2 Laboratorio de Investigación en Cáncer. Universidad Manuela Beltrán, Avenida Circunvalar No. 60-00, Bogotá 110231, Colombia; hugo.segura@umb.edu.co (H.S.-P.); lorena.nieto@umb.edu.co (D.N.-M.); catalina.ballesteros@umb.edu.co (A.V.-B.); diana.munoz@umb.edu.co (D.M.-F.); 3 Centre of Polymer Systems, University Institute. Tomas Bata University in Zlín, Trida Tomase Bati 5678, Zlin 76001, Czech Republic; munster@cps.utb.cz

**Keywords:** scaffolds, chitosan, poly(vinyl alcohol), cell proliferation, cell differentiation

## Abstract

Scaffolds can be considered as one of the most promising treatments for bone tissue regeneration. Herein, blends of chitosan, poly(vinyl alcohol), and hydroxyapatite in different ratios were used to synthesize scaffolds via freeze-drying. Mechanical tests, FTIR, swelling and solubility degree, DSC, morphology, and cell viability were used as characterization techniques. Statistical significance of the experiments was determined using a two-way analysis of variance (ANOVA) with *p* < 0.05. Crosslinked and plasticized scaffolds absorbed five times more water than non-crosslinked and plasticized ones, which is an indicator of better hydrophilic features, as well as adequate resistance to water without detriment of the swelling potential. Indeed, the tested mechanical properties were notably higher for samples which were undergone to crosslinking and plasticized process. The presence of chitosan is determinant in pore formation and distribution which is an imperative for cell communication. Uniform pore size with diameters ranging from 142 to 519 µm were obtained, a range that has been described as optimal for bone tissue regeneration. Moreover, cytotoxicity was considered as negligible in the tested conditions, and viability indicates that the material might have potential as a bone regeneration system.

## 1. Introduction

Biomedical engineering is considered as an extension of chemical engineering towards biomaterials, and tissue engineering (TE) is one of its main branches [1]. TE aims to regenerate damaged tissues by combining cells from the body with highly porous scaffold biomaterials, which act as template for tissue regeneration and guide the growth of new tissue [2]. A very promising potential in creating biological alternatives for harvested tissues, implants, and prosthesis has been reported by TE [3] and the ability to aid treatment of numerous clinical situations, including spinal fusion, joint replacement, fracture nonunion, and pathological loss of bones, among other conditions [4]. Within this frame, bone tissue as a cornerstone of the muscle-skeletal system, is a hard, calcified connective tissue with functions of protection and mobility of the body, storing calcium as well as mesenchymal and hematopoietic stem cells [5]. Nonetheless, bones are susceptible to damage, specifically caused by fractures, tumors, osteoporosis, and other sources of trauma, and although bones have certain self-regenerative properties, there are conditions in which complete tissue restoration cannot be achieved exclusively by internal processes [6].

Over the last ten years, remarkable progress has been made in the development of surgical techniques for bone reconstruction. Synthesis of functional bone using combinations of cells and bioactive factors is considered as a promising approach towards bone regeneration, undoubtedly creating the possibility of tissue regeneration and repair [7]. However, TE has studied a technique able to supply the needs for supporting and proliferating regenerative cells: scaffolding [8]. This technique has been extensively studied in bone tissue regeneration and seeks to mimic bone extracellular matrix architecture.

Scaffolds aim at the creation of microenvironments for cell adhesion, proliferation and differentiation [9,10,11] and they play a key role in bone TE providing a 3-dimensional environment for cell seeding as well as filling bone defects while providing mechanical competence during bone regeneration [12]. Currently, there are different methods for synthesizing scaffolds and each method provides unique features to the sample and defines its final use [13,14,15,16,17]. Thus, polymers come as great candidates for scaffold fabrication due to their ability to be used in the aforementioned methods.

A large variety of polymers has been used in manufacturing scaffolds, both natural and synthetic. Natural ones provide outstanding properties to scaffolds, from fostering active interactions with cells to hampering immune response from the host, whereas synthetic polymers display better mechanical properties. Poly(l-lactic acid) (PLLA) [18], poly(glycolic acid) (PGA) [19], poly(caprolactone) (PCL) [20], and poly(lactic-co-glycolic) acid (PLGA) [21] are currently the most common materials used as scaffolds for bone regeneration [22,23]. Within natural polymers, chitosan (CH) deserves special attention because this polysaccharide exhibits antibacterial, antifungal, mucoadhesive and analgesic properties [24]. In addition, CH can be degraded by a set of human enzymes and is biocompatible under certain conditions [25]. Innumerable reports in which CH is used in scaffolds for bone regeneration can be found in literature [24,26,27,28,29,30,31,32,33] due to the fact that CH facilitates proliferation of osteoblast cells and helps to create mineralized bone matrix. While CH exhibits suitable biological properties for bone tissue regeneration, this type of material needs additional treatment, such as crosslinking, to be able to withstand the physical conditions required by a bone implant [25,34]. However, by blending synthetic polymers with natural ones, scaffolds might exhibit better mechanical stability [35]. In this frame, polyvinyl alcohol (PVA) represents an outstanding candidate for bone TE due to its biocompatibility and its low cytotoxicity as it has been demonstrated in previous studies [36,37,38,39,40,41]. Moreover, bioactive compounds are often used in TE to facilitate interactions between scaffolds and cells, promoting thus adhesion, proliferation and differentiation [42,43]. Therefore, hydroxyapatite, a major inorganic component in bone tissue [44], comes as a very promising bioactive compound as it may provide the material the ability to mimic natural bone functionality and ultimately promoting osteoconductivity [45]. Accordingly, several studies on CH-HA have been carried out, evidencing the ability of HA to improve bioactivity [44,45,46,47,48]. CH, PVA, and HA have been investigated into the bone regeneration frame separately and copious literature support the importance of the materials. Nonetheless, the combination of the mentioned materials appeared in only 5 publications in the last year according to Web of Science. Therefore, the system is still an unexplored niche, and it has a notably potential and innovative opportunity for bone tissue engineering.

Considering the aforementioned reasons, a crosslinked and plasticized PVA-CH-HA system rises as a potential candidate for bone tissue engineering and therefore, this research is aimed to determine the potential of the system in bone regeneration through biological, mechanical and physicochemical characterization.

## 2. Results and Discussion

Scaffold characterization consists of collecting information which might elucidate how the material will perform under specific conditions. In this matter, techniques such as degree of swelling, solubility degree, FTIR, DSC, SEM, mechanical characterization and cell culture will provide sufficient and relevant evidence about how the material behaves and how the material might interact with its own components as well as with the surroundings.

### 2.1. Degree of Swelling (DS) and Solubility Degree (SD)

As scaffolds are designed to be subject to high humidity conditions, swelling and solubility degree are fundamental parameters to be studied. Water absorption is an imperative feature for scaffolds as the ability to conduce and store water through a porous network is essential to achieve proper cell signaling and nutrition [45]. The mechanism of swelling requires the polymeric chains to be able to soak up water molecules across the matrix. To do so, reversible interactions such as hydrogen bonds must be fostered [25].

**Figure 1:**
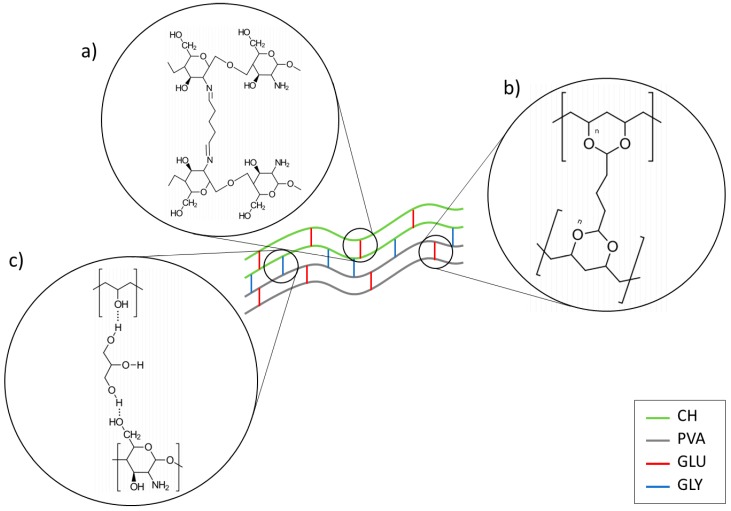
Schematic representation of interactions between CH, PVA, GLU, and GLY. (**a**) CH crosslinked by GLU. (**b**) PVA crosslinked by GLU. (**c**) Hydrogen bonding between CH and PVA with GLY.

Significant differences were observed in both, crosslinked and plasticized (CPS) and non-crosslinked and non-plasticized samples (nCPS). Although considerable modifications in volume and fluctuations in shape were presented in both CPS and nCPS, the former were able to resist manipulation without fracture in contrast to nCPS which became fragile, yielding difficult manipulation and eventual fracture. CPS absorbed from 600 to 1700 times their own weight whereas nCPS presented notably higher values (Table 1). This behavior might be explained by three approaches which are depicted in Figure 1. In the first one, crosslinking between glutaraldehyde (GLU) and -NH_2_ reduces the concentration of amino groups, leading to a reduction of interactions with water [49,50]. The second approach indicates that a lesser content of hydroxyl groups, due to PVA crosslinking, becomes available [51]. And finally, interactions between hydroxyl groups of PVA and amino or hydroxyl groups of CH reduces the number of interactions between -OH groups and water [52], reducing the overall hydrophilicity of the system. Since the hydrophilicity dominates the behavior of the scaffolds in this test, a higher concentration of CH increases DS. Although CH amine functional groups are more reactive to GLU than hydroxyl groups of PVA [53,54] the low concentration of the crosslinker did not dramatically reduce the presence of hydrophilic groups and the system exhibits a CH-like behavior, dominating the DS of the system. Furthermore, a reduction in the crystalline phase (see DSC) caused by CH produces a augmentation in the capacity of the material to absorb water and therefore, a rise in the DS [51].

On the other hand, nCPS did not reduce the availability of hydrophilic groups which allowed water molecules to create non-covalent bonds. In nCPS, PVA molecules are entangled with CH (physical interactions) whereas in the CPS ones, chemical crosslinking was achieved, forming covalent bonds among chains, fixing and reducing polymer mobility, which resulted in the lower DS [54].

There is an intimate relation between DS and SD. In both cases, the interactions with water are responsible for the observed behavior. It has been reported that solubility of PVA depends on the degree of hydrolysis, the molecular weight, and the tendency to form hydrogen bonding in aqueous solutions. In this matter, any alteration of those factors will have repercussions on the solubility [51]. Although CPS have more free space and therefore water could go through the system, the presence of GLU attaches the polymer chains and hydration might be difficult. Consequently, CPS exhibit lower SD than nCPS. The chemical bonds created during the treatment gives the material resistance and stability. Indeed, the lesser hydroxyl groups reduced the affinity for water leading to a reduction in the swelling as well as to the solubility. Although, CH is not soluble in water, its -NH_2_ groups allow the polymeric chains to partially attach themselves to water molecules, increasing thus the solubility of the material. It can be expected that a higher PVA concentration supposes higher levels of solubility. Nonetheless, the combination of PVA with CH generates a system with lower SD than PVA alone, possibly due to intermolecular interactions between polymeric chains instead of water interactions [52]. It can be inferred then that CPS and nCPS with a high presence of CH lead to the material mechanical stability and a high capacity to absorb and transport water.

### 2.2. Fourier Transform Infrared Spectroscopy (FTIR)

**Figure 2:**
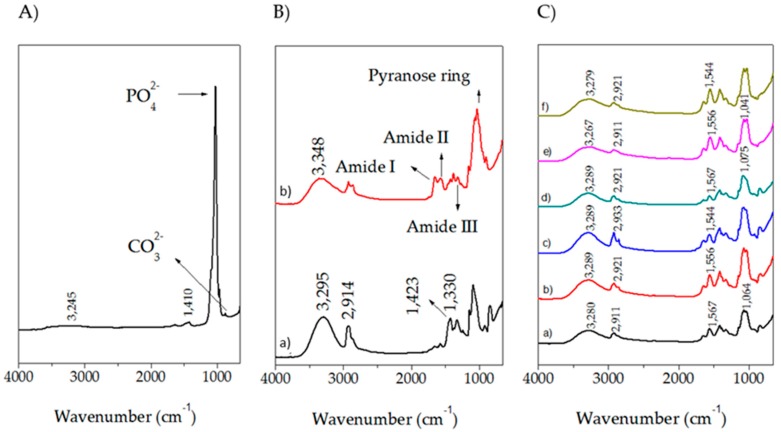
Collection of the obtained FTIR spectra. (**A**) Hydroxyapatite; (**B**) (a) PVA and (b) CH; (**C**) Prepared scaffolds (a) CH:PVA 1:1 GLU-GLY; (b) CH:PVA 1:1; (c) CH:PVA 1:3 GLU-GLY; (d) CH:PVA 1:3; (e) CH:PVA 3:1 GLU-GLY; (f) CH:PVA 3:1.

FTIR is a crucial technique for polymer characterization and it provides information about crosslinking between polymeric chains. Figure 2 shows the obtained spectra for all the prepared samples and pure components. The spectra were vertically moved for better clarity. HA exhibits vibrations located at 630 and 3570 cm^−1^ which correspond to hydroxyl ions [55] as well as signals located at 1023 and 962 cm^−1^ that belong to PO_4_^2−^ groups, and one peak at 874 cm^−1^, which is associated to CO_3_^2−^ [56,57].

The spectrum for PVA shows a strong broad absorption centered at 3295 cm^−1^, associated to hydroxyl functional groups. Saturated C–H stretching is manifested in the band at 2914 cm^−1^ and the band located at 1423 cm^−1^ corresponds to –CH_2_– bending. No signals around 1700 cm^−1^ were visible, which indicates the absence of acetate groups, caused by the high percentage of hydrolysis of PVA. The bands at 1415 and 1330 cm^−1^ can be attributed to combination frequencies of CH and OH [58]. The CH_2_ rocking band at 928 cm^−1^ and a peak at 848 cm^−1^ are associated with C–O stretching and these results are in a good agreement with other reports [59,60,61,62].

The CH spectrum exhibits a broad signal centered at 3348 cm^−1^ corresponding to OH functional groups and NH group stretching vibrations. The symmetric, and asymmetric –CH_2_– stretching occurred in the pyranose ring are located at 2920, 2880, 1430, 1320, 1275 and 1245 cm^−1^ [54,63,64]. The bands located at 1650, 1586 cm^−1^, and 1322 cm^−1^ are ascribed to amide I, amide II, and amide III respectively as well as the saccharine-related signals at 1155 and 900 cm^−1^ [49,54,65,66,67]. The peak at 1030 and 1080 cm^−1^ indicates the C–O stretching vibration.

The effect of the plasticizer (glycerol, GLY) on the samples can be evidenced in the slightly rise in the signal corresponding to hydroxyl groups. Nonetheless, the combination of both, GLY and GLU have contradictory consequences; on one side, the crosslinker diminished hydroxyl groups, but on the other side, the plasticizer increases them. For that reason, no clearly observable tendency is depicted in the spectra.

CPS presents an increase of the signal for imine bands as well as a drop on the amine caused by the nucleophilic addition of the amine from CH with the aldehyde [68]. Furthermore, blends of CH and PVA might produce carboxylic acid dimer which is manifested in the region of 1700–1725 cm^−1^ and are originated by the acetic acid used for dissolving CH. An evident decreasing of absorbance around 1550 cm^−1^ proves that crosslinking decreased the availability of NH_2_ groups. Simultaneously, CPS spectra show lower absorbance at the –OH band because this group is consumed in the formation of C–O–C bonds [69]. Moreover, the absorption at 3300–3250 cm^−1^ related to OH and NH stretching vibrations broadened and shifted to a lower wave number with the increase of PVA, suggesting the formation of hydrogen bonds between CH and PVA [70].

A reduction of the intensities of bands in the range of 1500–1650 cm^−1^ was evidenced in samples where the CH content was diminished. Another observed feature is that the region from 3200 and 3500 cm^−1^ is wider in blends than in single components as a result of the overlapping signals from both hydroxyl and amino groups. The wavenumber of this peak has shifted toward lower values by increasing the PVA content in the samples, which indicates hydrogen bond formation between PVA and CH. The peak around 1560 cm^−1^ was a weaker N-H vibration and was depressed by increasing the PVA content in the samples. This is another indication of hydrogen bonding between PVA and CH chains [71].

### 2.3. Mechanical Characterization

**Figure 3:**
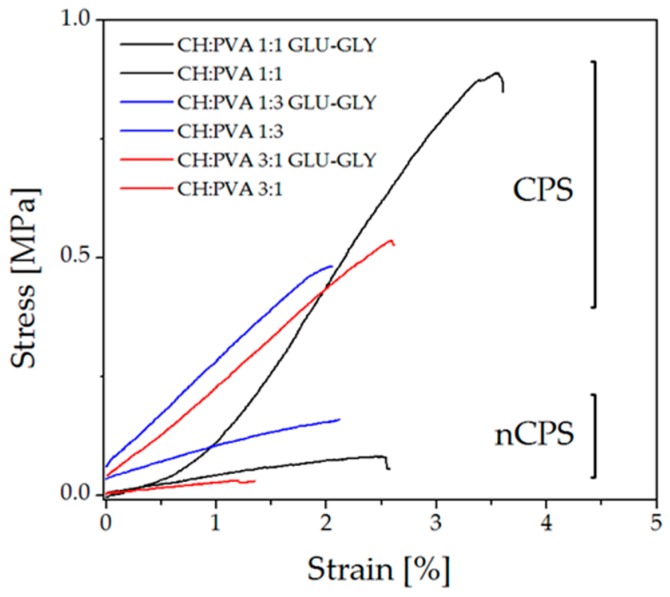
Tensile stress-strain curves for prepared samples.

Young’s Modulus (*E*), tensile strength (*TS*) and yield strength (*YS*) were chosen for evaluating how the material behaves under mechanical loads and the results are reported in Table 2. The first remarkable feature of the studied samples is that differences in Young’s Modulus are caused by the presence of the crosslinker and the plasticizer. As can be noticed, CPS display notably higher values than nCPS because GLU is chemically joined to both polymers reducing chain mobility. Nonetheless, as it was mentioned above (see swelling and solubility analysis), CH amine groups are more reactive to GLU than PVA, and therefore, the blend exhibits CH-like features. Indeed, by bonding polymer chains with GLU through Schiff bases and acetal bonds, the material improves its toughness. Therefore, CPS require more energy to be elongated and, ultimately, break. This behavior can be seen in Figure 3 where samples at >1% strain grouped in separate zones of the graph; CPS are found above nCPS along the Y-axis, enclosing a larger area under the curve than nCPS.

On the other hand, GLY can be considered as responsible for a higher *YS*. This may be caused by a reinforcement of the interactions between polymeric chains. GLY facilitates interactions between zones of the polymeric chains that were not crosslinked, increasing thus the number of interactions between them and its presence produces polymer-plasticizer interactions among the macromolecules to the detriment of polymer-polymer interactions and it causes a decrease of the hardness as well as Young’s modulus [62]. Moreover, the presence of GLU and GLY has an important effect on *TS*. GLY is responsible for a relevant rise in intermolecular forces by creating interactions between polymeric chains through hydrogen bonds, as was explained previously (Figure 1c). These interactions might have the effect of preventing chitosan from crystallizing, although does not significantly destroy the crystalline component of PVA [72]. Simultaneously, some fractions of the polymeric chains are joined together chemically by GLU. Consequently, *TS* is affected by these intermolecular interactions and bonds, increasing thus the tensile strength of the sample [73].

Polymer concentration is also a variable which affects the tested properties, *E*, *TS* and *YS.* As can be seen, the highest values were obtained for CH:PVA 1:1-GLU/GLY whereas a reduction of the tested parameters were obtained at the concentration of any of the two polymers changed. This behavior is in concordance with previous studies of CH and PVA interactions [74]. These evaluations indicate that CPS at equal concentrations of CH and PVA exhibit conditions to endure mechanical loads, which might indicate that under the tested conditions, the material will perform adequately as a candidate for bone tissue regeneration. Nonetheless, a higher concentration of CH is also appropriate for that purpose.

### 2.4. Differential Scanning Calorimetry

**Figure 4:**
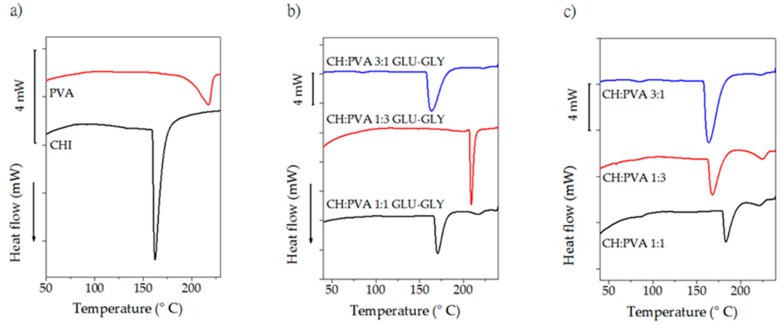
Thermograms for the studied materials. (**a**) Pure polymers; (**b**) CPS; (**c**) nCPS.

Thermograms for pure components and prepared scaffolds are shown in Figure 4. Glass transition temperature (T*_g_*) and melting point (T*_m_*) (Table 3) for PVA are located at 72 °C and 218 °C respectively, results that have been reported for other authors [51,75,76]. CH, on the other hand, exhibits an endothermic transition between 100–120 °C which is attributed to evaporation of residual water [63]. A sharp peak centered at 161 °C is not a manifestation of T*_m_* since a recrystallization peak did not appear neither in the second cooling nor in the second heating scan. Therefore, the peak presumably results from the dissociation process of interchain hydrogen bonding [77].

Several attempts at obtaining T*_g_* data were carried out and there was no unanimity in the reported values so DSC might not be an appropriate technique for detecting T*_g_* since CH is a semi-crystalline polymer [78]. Nonetheless, it has been established that the degree of deacetylation (75–85% in this case) has not influence in the T*_g_*, and α-relaxations occurred at 140–150 °C which might be a reliable value for T*_g_* [79], transition which is hidden by the broad peak in the same region.

Important features are observable in the thermograms for the CPS and nCPS. Detecting T*_g_* for CH is problematic, and the same difficulty was observed for that transition in the blends under the studied conditions, presumably for the overlapping of the transitions occurred in both CH and PVA. In the case of CPS, it can be assumed that the strong interactions such as inter-molecular hydrogen bonding inhibit the movement of the molecular chain sections and an increase of the T*_g_* value might be achieved [80,81]. Furthermore, an increment in CH concentration causes higher values for T*_g_* in the blend in comparison to PVA, because polymer interactions through the hydrogen bonding formation causes an effective increase in the mean molecular weight [82]. Despite the impossibility to detect T*_g_*, all the prepared scaffolds should show a unique T*_g_* which is a manifestation of the compatibility of PVA and CH [83]. On the other hand, the Tm of the blends is reduced as the concentration of CH rises and a depression of T*_m_* is considered as a measure of the blend compatibility [42,83]. The addition of GLY led to more hydrogen bonds, which has an impact on the structural order of the matrix and therefore a reduction in T*_g_* should be evident [84]. Nonetheless, apart from the low concentration of GLY and GLU, the presence of PVA is determinant in the thermal behavior of the scaffold. The T*_m_* estimated for pure PVA is 218 °C which in combination with CH was notably reduced, yielding to a sharp and deep endothermic peak.

It might be expected that the crystalline nature of PVA was decreased by the addition of CH, which is in concordance to the DS and FTIR data [42]. Indeed, crosslinking limited the mobility of the polymers and suppressed the crystallites growing (in the case of PVA), disfavoring crystallization. Additionally, the reduction of crystallinity is an indicative of a lower extent of the confinement of molecular chain segments, and the interpenetration and entanglement of GLU-CH with PVA. 

### 2.5. Scanning Electron Microscopy

**Figure 5:**
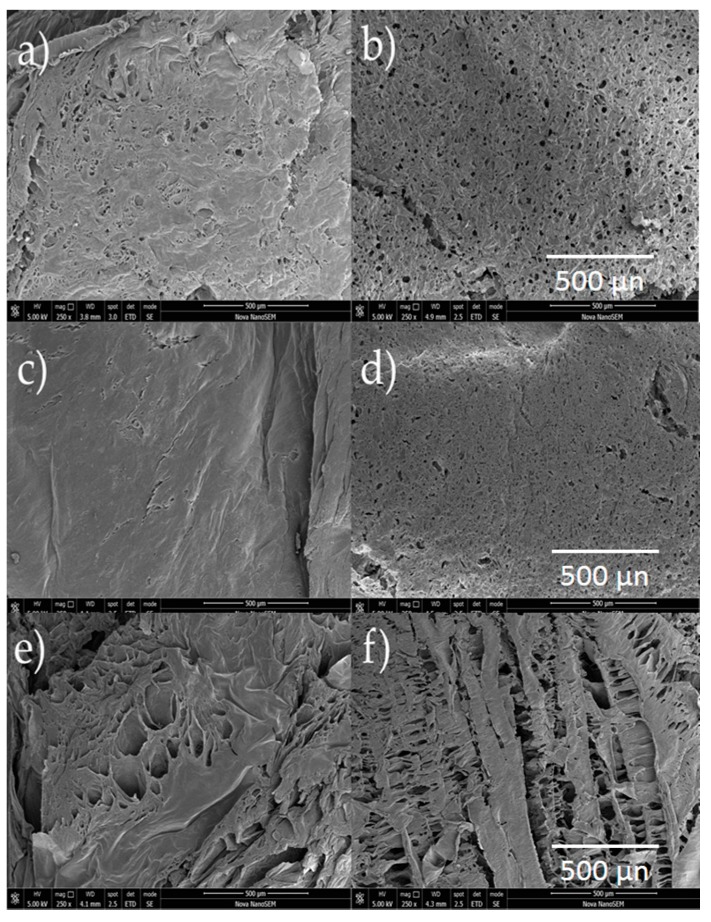
Surface SEM images for CPS (**left**) and nCPS (**right**) at 250×. (**a**) CH:PVA 1:1 GLU-GLY; (**b**) CH:PVA 1:1; (**c**) CH:PVA 1:3 GLU-GLY; (**d**) CH:PVA 1:3; (**e**) CH:PVA 3:1 GLU-GLY; (**f**) CH:PVA 3:1.

SEM images provides information about structural and morphological features of the scaffolds. The first observable characteristic is that morphology varies depending on both the presence of GLU-GLY as well as the concentration of CH and PVA. As can be seen in Figure 5, there is a reduction in pore size as a consequence of chemical crosslinking in most examples, except in CH:PVA 3:1 in which average pore size increased four times when crosslinked. Conversely, a higher concentration of PVA led to a lower pore size [85]. Porosity decreased in CPS 1:3 compared to nCPS 1:3 suggesting that CH might act as a factor to stop the porosity size and void percentage of crosslinked-PVA-containing samples from decreasing. CH 3:1 PVA samples showed a higher pore size and void percentage than CH 1:3 PVA indicating that CH controls pore formation within the scaffold (Table 4). Equal concentration of each polymer showed a combination of both behaviors. Polymer concentration did not play a significative role in fiber formation. Lastly, it is reasonable to believe that void percentage and pore size is affected by freeze-drying and therefore, modifying vacuum pressure and time of might produce scaffolds with different pore size [40].

**Figure 6:**
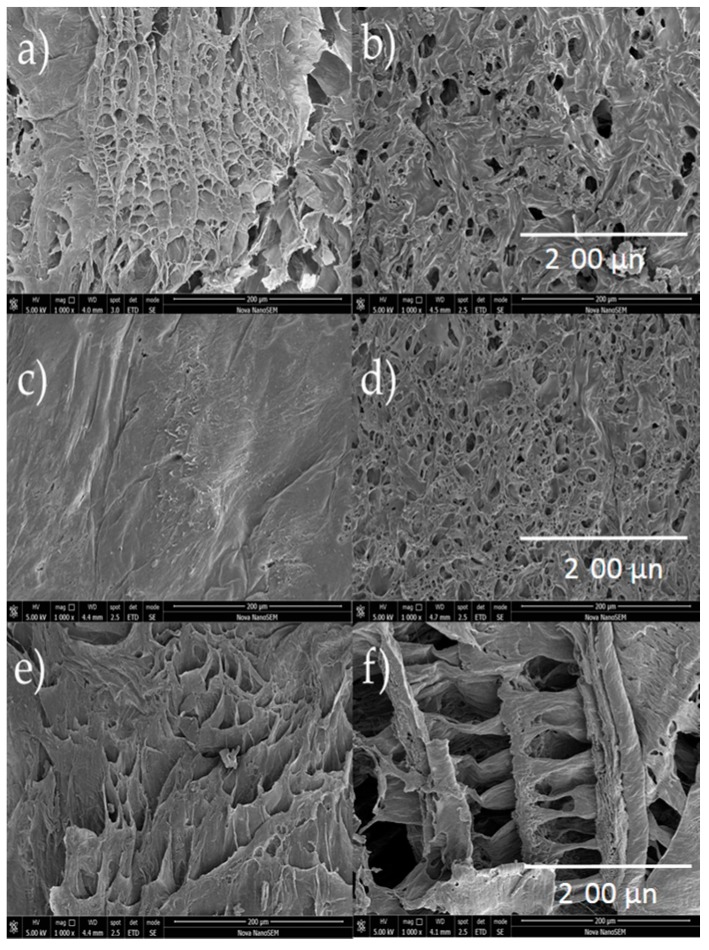
SEM images of surface for CPS (**left**) and nCPS (**right**) at 1000×. (**a**) CH:PVA 1:1 GLU-GLY; (**b**) CH:PVA 1:1; (**c**) CH:PVA 1:3 GLU-GLY; (**d**) CH:PVA 1:3; (**e**) CH:PVA 3:1 GLU-GLY; (**f**) CH:PVA 3:1.

Another crucial parameter for pore formation is viscosity. The original solution of CH is too viscous, and the sample does not mix homogeneously, therefore the pore formation and size is reduced during the freeze-drying process. Consequently, the pore regularity is affected [86] and samples with lower CH concentration present less regular pores (Figure 6). Indeed, CPS 1:1 show higher pore size regularity and distribution while nCPS 1:1 exhibit a semi-porous surface, suggesting low pore interconnectivity. This behavior was consistently observed in all CPS and nCPS.

**Figure 7:**
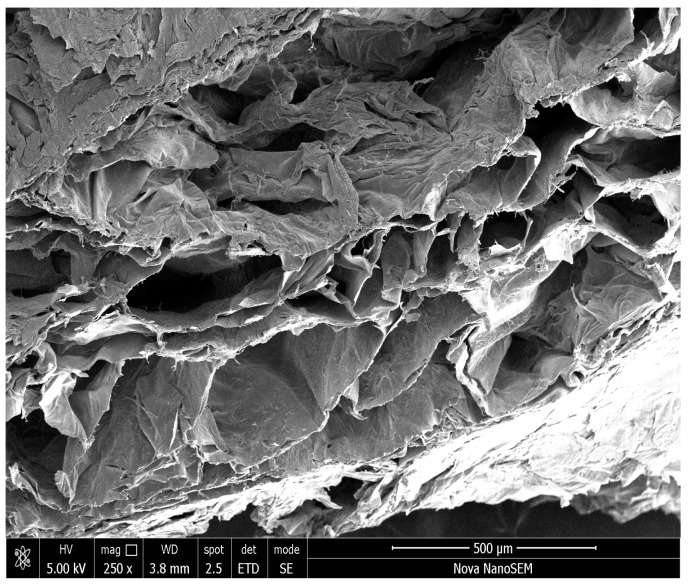
Bulk SEM images at 250× of CH:PVA 3:1 GLU-GLY.

Despite all the morphological features exhibited by CPS and nCPS, CH:PVA 3:1 GLU-GLU (Figure 7) seems to be adequate for bone tissue engineering. The data obtained during the evaluation of pore size revealed uniform pore formation with diameters ranging from 142 to 519 µm, a range that has been described as optimal for bone tissue regeneration [87] and the pore structure obtained is suitable for bone tissue engineering by exhibiting a homogeneous macro-porous network with open pores and some interconnectivity. Such hierarchical structure has potential application for future bone tissue in-growth, nutrient delivery and vascularization [88].

### 2.6. Cell Culture

**Figure 8:**
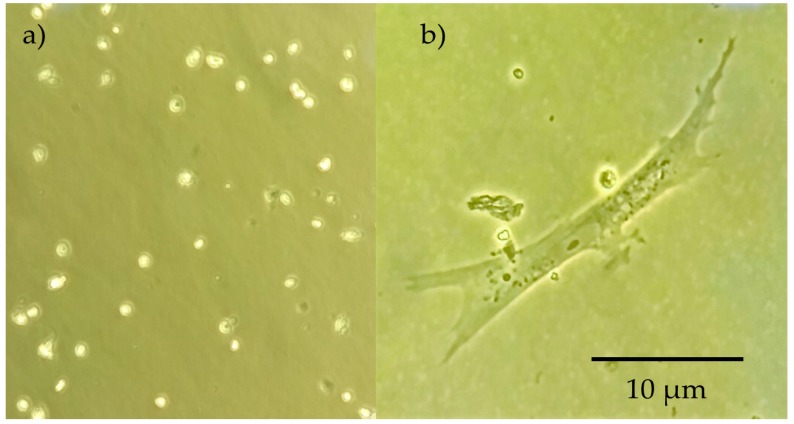
(**a**) Trypsinized osteoblastic cells. (**b**) Osteocyte cell found in the surroundings of sample CH 1:3 PVA + GLU/GLY.

Cell culture was performed in triplicate for all the prepared scaffold during 17 days, and cell differentiation was evaluated through morphologic analysis. Initial conditions of trypsinized osteoblastic cells are shown in Figure 8. After three days of culture, a higher degree of differentiated cells were present in CPS, exhibiting the formation of osteocytic canaliculi (Figure 8b) which suggest proper cell signaling through osteocyte gap junctions [89]. Furthermore, the characteristic star-like morphology for osteocyte are visible in the image, suggesting that the process from osteoblast to osteocyte has been carried out. These cytoplasmic projections allow to the cell to communicate with surrounding osteocytes using gap join [90]. Proliferation continued during the first 5 days of the experiment. Later, it was not possible to evaluate it, as cells migrated inside the scaffolds and for observation it was needed to destroy the scaffold. Therefore, cell activity was evaluated through colorimetric observations of culture media and it was constant until 7 days of culture.

**Figure 9:**
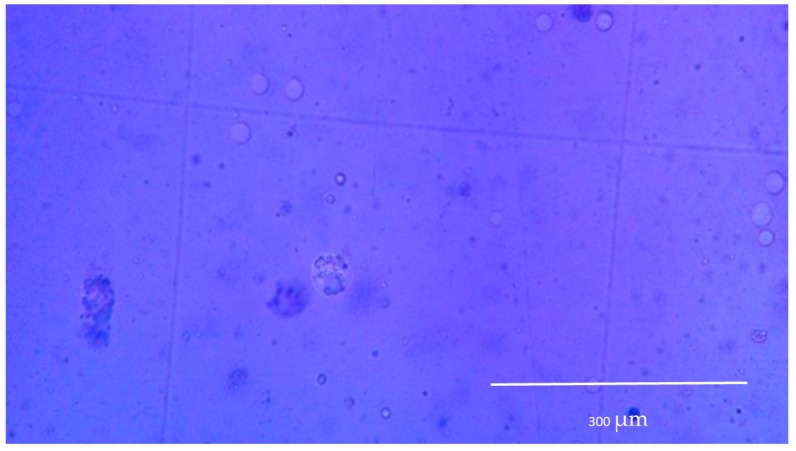
Cell culture after 5 days.

Cell viability was assessed using the Trypan Blue Exclusion Method during the first days of the experiment. In this frame, Figure 9 exhibits cell with rigid membrane as well as translucid cells which might indicate that the material performed appropriately in terms of viability. On the other hand, dead cells appeared, and membrane fragmentation is presented, although it is important to emphasize that its number is considerably lower. Moreover, shattering did not affect substantially cell viability, and although turbidity might affect the adequate nutrients path, signaling was kept even in these adverse conditions. Another indicator for viability is that characteristic morphological changes occurs. In a viable state, the lacuna and the osteocyte inside are both star-shaped [91].

During the last days of the experiment, cell activity was significantly reduced until no change in culture media was observed, suggesting high mortality rate. Supporting this theory, cell detritus appeared in culture media. This fact might have been produced by early degradation of the sample, hindering continuous cell signaling. Nonetheless, the concentration of non-crosslinked GLU contained within the sample was too low and even if it was released as samples degraded, it might not have contributed to cytotoxicity.

## 3. Materials and Methods

### 3.1. General Information

Poly(vinyl alcohol) (PVA) (M*w* = 130,000 g mol^−1^) with 99% of hydrolysis, chitosan (CH) of medium molecular weight with deacetylation degree of 75–85%, glutaraldehyde (GLU) as aqueous solution grade II at 25%, hydroxyapatite (HA), ethanol 99.8%, Dulbecco’s Modified Eagle’s Medium–high glucose (DMEM high glucose culture medium), antibiotic solutions (penicillin-streptomycin, and amphotericin B), and Trypan blue were acquired from Sigma-Aldrich (Bogotá, Colombia). Glycerol (GLY) 99.5% was purchased from (Bogotá, Colombia). Fetal Calf Serum (FCS) was provided by Microgen (Bogotá, Colombia). None of the reagents were subject to further processing.

The scaffolds were prepared as follows: A 3% *w*/*v* acid solution of CH was prepared by the addition of the polymer to acetic acid at 0.2 M under mild manual stirring. Once the polymer was partially dissolved, the solution was stirred in a shaker for 24 h using a Unimax 1010DT instrument (Heidolph, Schwabach, Germany). On the other hand, an aqueous solution of PVA at 3% *w*/*v* was prepared in deionized water at 70 °C under vigorous magnetic stirred using a magnetic stirred (Model SP131325Q, Thermo-Scientific, Shangai, China). HA was then added to the previous solutions at 5% *w*/*w* related to the total amount of the polymer, and the new blends were stirred until a homogeneous system was formed. Two sets of polymeric solutions were prepared in three different volume ratios (1:1, 1:3 and 3:1). GLU and GLY at 0.001% *w*/*w* and 2.5% *w*/*w* related to the total amount of the polymer were added respectively to the first set as cross-linker and plasticizer respectively, and the blends were stored at 4 °C to allow crosslinking reactions. The second set was maintained as a control group. The prepared solutions were poured in molds to create disks and layers and frozen at −80 °C before freeze-drying process. Finally, the samples were freeze-dried at −40 °C and 3 mbar for 48 h (Super Modulyo manifold lyophilizer, Edwardsm, Wilmington, DE, USA) and kept at −4 °C until testing.

### 3.2. Degree of Swelling (DS) and Solubility Degree (SD)

Gravimetric method was used to obtain degree of swelling and solubility degree. Disk specimens were dried in a desiccator for 24 h to obtain dry weight (W1). Then, the samples were immersed in deionized water at 20 °C for several periods (2, 4, 6, 8, 10, 15, 20, 30, 60, 90 min and 24 h). At the end of each period excess of water was removed from the surface with filter paper and specimens were weighed again. The last measure (24 h) was considered as the swelling limit (W*2*) because in that period the equilibrium was reached. Finally, samples were dried until constant weight (W*3*). Degree of swelling (DS) and solubility degree (SD) were determined using Equations (1) and (2), respectively. The test was performed with three specimens for each sample (n = 3) to obtain statistically significant data:

DS = [(W_2_ − W_1_)/W_1_] × 100 (1)

SD = [(W_1_ − W_3_)/W_3_] × 100 (2)

### 3.3. FTIR-ATR Spectroscopy

Spectra for pure CH, PVA, HA and all blends with and without additives were obtained using a Nicolet iS5 spectrometer (Thermo Fisher, Waltham, MA, USA) equipped with attenuated total reflectance (ATR) accessory utilizing the Zn-Se crystal. Each spectrum represents 64 co-added scans referenced against an empty ATR cell spectrum. The spectra range was from 4000 to 650 cm^−1^ with a resolution of 1.92 cm^−1^.

### 3.4. Mechanical Characterization

Young’s modulus, tensile strength, and Yield strength were evaluated in tensile mode and tested on six specimens per sample using a rectangular test specimen specified in ASTM-F-2150-01/05 using a M350-CT Testometric tester (Lincoln Close, Rochdale, UK). The rate was 2 mm/min and the thickness of the samples was measured by a micrometer with the accuracy of 0.01 mm.

### 3.5. Differential Scanning Calorimetry (DSC)

Calorimetric measurements were carried out in a DSC 1 calorimeter, Mettler Toledo (Zurich, Switzerland), under nitrogen flowing at a rate 30 mL min^−1^. The specimens were pressed in unsealed aluminum pans. Glass transition temperature (T*_g_*) and melting temperature (T*_m_*) were obtained, and the former was determined as the midpoint temperature by standard extrapolation of the linear part of DSC curves whereas the later as the maximum value of the melting peak. The samples were cooled down by air at an exponentially decreasing rate. The heating of the cycle was performed from 25 to 240 °C at a rate of 20 °C/min. The relative crystallinity (*Xc*) was estimated from the endothermic area using Equation (3), where ΔH_f_ is the measured enthalpy of the fusion from DSC thermograms and ΔH_f_^0^ is the enthalpy of fusion for 100% crystalline PVA (138.6 J g^−1^) [92]:

Xc = ΔH_f_/ΔH_f_^0^ (3)

### 3.6. Scanning Electron Microscopy

Micrographs of the prepared samples were taken by a Nova NanoSEM 450 (FEI, Brno, Czech Republic) scanning electron microscope with Schottky field emission electron source operated at acceleration voltage ranging from 200 V to 30 kV and low-vacuum SED (LVD) detector. A coating with a thin layer of gold was performed by a sputter coater SC 7640 (Quorum Technologies, Newhaven, East Sussex, UK). Pore size and surface void percentage were determined using the Fiji biological image analysis software [93]. Average pore sizes were obtained by measuring ten randomly selected pores from SEM images and void percentage was calculated by processing SEM images to obtain 8-bit (black and white) micrographs of the surface of the scaffold and then calculating a percentage of void representing pixels using Equation (4), where V_p_ is the number of void pixels (black) and S_p_ (white) is the number of surface pixels:

Void % = V_p_/(V_p_ + S_p_) (4)

### 3.7. Cell Culture

Samples were sterilized using an ethanol series and neutralized in phosphate-buffered saline PBS at 4 °C which also worked as a progressive rehydration of the samples to avoid sudden and excessive swelling (see Section 2.1). Neutrality was confirmed by incubating the samples in DMEM culture media supplemented with 5% FBS, and 1% of antibiotic solution at 37 °C, 5% CO2 for 24 h to check color and pH changes of the media. Osteoblasts were obtained from a primary culture contained from an 8-day bird embryo; cells were washed in a PBS with antibiotics solution and subject to trypsinization for 30 min to separate them from the bottom of the flask to obtain a subculture. Once the subculture was confluent, cells were re-trypsinized and suspended for cultivation into the prepared scaffolds. Viability was assessed using the Trypan Blue Exclusion Method and cells were seeded at the same conditions of the initial culture. Cell culture was performed for 17 days, changing culture media and observing cells through the inverted microscope every three days.

### 3.8. Statistical Analysis

All experiments with quantitative measurements were performed in triplicate, except for mechanical testing where n = 6. All experimental values were expressed in form of average ± standard deviation. Results were statistically compared using two-way analysis of variance (ANOVA) with *p* < 0.05.

## 4. Conclusions

One of the main objectives for TE is the design of 3-dimensional materials able to mimic the bone extracellular matrix and ultimately allow bone cell adhesion, proliferation and differentiation. In this context, the prepared scaffolds were tested in order to elucidate how the material might behave in conditions which can be catalogued as similar for their possible performance as a candidate for bone regeneration. Individually, PVA and CH have singular properties which demonstrate benefits in biomedical science. However, the combination of both result in a new material with enhanced mechanical and biological properties. Within this framework, it is possible to claim that PVA-CH polymeric scaffolds with ulterior polymeric treatment, exhibit potential for bone tissue regeneration. The collected information suggest that CPS are considerably more pertinent for withstand moisture conditions with an appropriate solubility resistance, property which can be considered as desirable for bone tissue regeneration. Furthermore, the material proves to be suitable for osteoblastic colonization, differentiation, and proliferation within a range of two weeks, which is an indicative of negligible level of cytotoxicity. The addition of GLU, GLY and HA to the polymeric solutions produced a well porous interconnected material which favors cell growth; CH is determinant in pore formation as a higher concentration produces a higher uniformity and distribution of porosity. Variations in concentration of the polymers indicated that CPS with a CH:PVA 3:1 ratio exhibit a higher potential in tissue engineering and should be subjected to further analysis to determine to a full extent its limits in osteoblastic proliferation and ultimate bone regeneration.

## Figures and Tables

**Table 1:** Degree of swelling and solubility degree for CPS and nCPS.

Type of Sample	Ratio (*wt*/*wt*)	Degree of Swelling after 24 h [%] (DS)	Average Weight Loss [%] (SD)
CPS	CH:PVA 1:1-GLU/GLY	1004.44 ± 17.70	9.44 ± 1.20
	CH:PVA 1:3-GLU/GLY	621.97 ± 61.12	9.38 ± 0.40
	CH:PVA 3:1-GLU/GLY	1766.18 ± 347.95	17.82 ± 16.21
nCPS	CH:PVA 1:1	5010.20 ± 158.78	49.57 ± 1.01
	CH:PVA 1:3	2633.24 ± 176.38	34.31 ± 3.24
	CH:PVA 3:1	9883.13 ± 182.25	N/A

**Table 2:** Mechanical properties for the prepared scaffolds.

Type of Sample	Proportion	Young’s Modulus [MPa]	Tensile Strength [MPa]	Yield Strength [MPa]
CPS	CH:PVA 1:1-GLU/GLY	37.26 ± 10.35	0.96 ± 0.20	0.76 ± 0.34
	CH:PVA 1:3-GLU/GLY	27.29 ± 23.33	0.61 ± 0.41	0.57 ± 0.52
	CH:1 PVA 3:1-GLU/GLY	24.84 ± 9.83	0.67 ± 0.36	0.61 ± 0.35
nCPS	CH:PVA 1:1	4.70 ± 2.41	0.08 ± 0.04	0.03 ± 0.02
	CH:PVA 1:3	7.42 ± 1.10	0.23 ± 0.07	0.07 ± 0.02
	CH:PVA 3:1	2.61 ± 0.66	0.08 ± 0.05	0.02 ± 0.01

**Table 3:** Thermal characteristics of the studied samples.

Type of Sample	Ratio	Temperature (°C)		Enthalpy ΔH_f_ (mJ·g^−1^)	Crystallinity (%)
		T*_g_*	T*_m_*		
Pure polymers	CHITOSAN	-	-	-	-
	PVA	78	218	112	81
CPS	CH:PVA 1:1-GLU/GLY	-	170	67	48
	CH:PVA 1:3-GLU/GLY	-	210	125	90
	CH:PVA 3:1-GLU/GLY	-	160	266	-
nCPS	CH:PVA 1:1	-	185	69	49
	CH:PVA 1:3	-	165	108	78
	CH:PVA 3:1		162	224	-

**Table 4:** Obtained pore size for the evaluated scaffolds.

Type of Sample	Ratio	Pore Size (µm)	Void Percentage (%)
CPS	CH:PVA 1:1-GLU/GLY	11.7 ± 5.1	40.86
	CH:PVA 1:3-GLU/GLY	0.6 ± 0.2	6.48
	CH:PVA 3:1-GLU/GLY	331.5 ± 111.8	70.22
nCPS	CH:PVA 1:1	25.5 ± 9.0	4.95
	CH:PVA 1:3	11.9 ± 4.3	30.22
	CH:PVA 3:1	74.5 ± 32.4	78.28
